# Women's Perspectives on Influencers to the Utilisation of Skilled Delivery Care: An Explorative Qualitative Study in North West Ethiopia

**DOI:** 10.1155/2020/8207415

**Published:** 2020-02-10

**Authors:** Biruhtesfa Bekele Shiferaw, Lebitsi Maud Modiba

**Affiliations:** Department of Health Studies, University of South Africa, Pretoria, South Africa

## Abstract

Skilled attendance at birth is widely regarded as an effective intervention to reduce maternal and early neonatal morbidity and mortality. However, many women in Ethiopia still deliver without skilled assistance. This study was carried out to identify factors that influenced or motivated women to give birth in a health facility in their previous, current, and future pregnancies. This descriptive explorative qualitative study was conducted in two districts of West Gojjam zone in North West Ethiopia. Fourteen focus group discussions were conducted with pregnant women and women who gave birth within one year. An inductive thematic analysis approach was employed to analyze the qualitative data. In this study, two major themes and a number of subthemes emerged from the focus group discussions with the study participants. The factors that influenced or motivated women to give birth in health facility in their previous, current, and future pregnancies include access to ambulance transport service, prevention of mother to child HIV transmission service, referral service, women friendly service, and emergency obstetric services, good interpersonal care from health workers, and fear and experience of obstetric danger signs and complications. In addition, reception of information and advice on importance of skilled delivery care and obstetric danger signs and complications from health workers, use of antenatal care, previous use of skilled delivery care, ensuring wellbeing of parturient women and newborns, and use of emergency obstetric care were also identified as influencers and motivators for health facility childbirth in previous, current, and future deliveries. Increased understanding of the factors that influenced or motivated women to deliver in facilities could contribute to developing strategies to improve the uptake of facility-based maternity services and corresponding declines in maternal morbidity and mortality.

## 1. Introduction

Despite a remarkable decline in maternal mortality ratio (MMR) observed globally between 1990 and 2015, 303,000 maternal deaths were reported in 2015. Of these, developing regions accounted for 99% of the estimated maternal death, with sub-Saharan Africa alone accounting for about 66% of the estimated maternal death [1]. The maternal mortality ratio in Ethiopia had also substantially reduced from 871 maternal deaths/100,000 live births in 1990 to 421 maternal deaths/100,000 live births in 2016. However, this figure is still very high [2]. More than half of these maternal deaths worldwide were attributable to haemorrhage, hypertensive disorders, and sepsis followed by abortion, embolism, complication of anesthesia, and conditions of peripartum cardiomyopathy [3, 4].

Childbirth and its process are one of the most significant rites of passage to a woman [5]. The time of childbirth and immediately after birth is the most difficult period in a woman's life particularly in developing countries [6]. Improving access to and use of skilled attendants at birth in a setting with emergency obstetric care and a strong referral system is the single most effective intervention to substantially reduce maternal mortality ratio [7, 8]. Although institutional delivery rate in Ethiopia has shown improvement between 2000 and 2016, the proportion of births attended by a skilled birth attendant was very low (26%) [2].

Several studies conducted in many developing countries identified multitudinous influencing or motivating factors for health facility childbirth albeit abounding body of evidence derived from quantitative researches. Sociodemographic and economic factors including higher maternal education [9–18], higher wealth quintile [9, 11, 15], and employed by occupation [11] were found to have a positive association with health facility childbirth. Antenatal care attendance [9–19], birth order [10, 14, 15], previous childbirth in health facility [17], closer distance to health facility and access to transport [15], known pregnancy complication [9], planning to give birth in health facility [14], and quality of care [15] were also the factors that influenced women to utilise skilled birth attendance service. Furthermore, a community-based cross-sectional study conducted in North West Ethiopia revealed that fear and occurrence of complications, getting better service, safe and clean delivery, being informed to deliver at health facility during antenatal care, antenatal care attendance, and positive attitude of health workers were the main reasons for choosing skilled delivery service [13].

One of the health-related targets of the sustainable development goals (SDGs) is to reduce the global maternal mortality ratio to less than 70 per 100,000 live births by 2030 [20]. As part of this endeavor, the government of Ethiopia has set a target of reducing maternal mortality ratio from 420 to 199 per 100,000 live births between 2015 and 2020. A set of high impact interventions and strategies including family planning, focused antenatal care, skilled birth service, early postnatal care, improved health facility coverage, and expansion of emergency obstetric service are being implemented in Ethiopia to reduce the maternal mortality [21]; however, the coverage and impact of these interventions and strategies was low [2].

This study identified factors that influenced or motivated women to give birth in a health facility in their previous, current, and future pregnancies. Increased understanding of the factors that influenced or motivated women to deliver in facilities could contribute to developing strategies to improve the uptake of facility-based maternity services and corresponding declines in maternal morbidity and mortality.

## 2. Methods

### 2.1. Study Setting

This study was an integral part of a PHD project with the ultimate aim of developing strategies to improve the uptake of skilled birth attendance services in North West Ethiopia [22]. This research was conducted in two districts of Amhara regional state administration. This region was chosen on account of the low coverage of skilled attendance at birth (27.1%) [2]. The two districts, Womberema and Burie zuria, are located in West Gojjam zone and purposively selected based on their performances with respect to skilled attendance at birth. Besides, Burie zuria district health office comprises of four primary health care units (PHCU) under its supervision, namely, Tiatia, Kuche, Alefa, and Dereqwua primary health care units and all of them were included in this study. Womberema district health office also consists of four primary health care units and, of these, three of them (Shendi, Koki, and Wogedade primary health care units) were included in this study. Furthermore, one kebele and one health centre were purposively selected from each of the primary health care units. A kebele is the smallest administrative unit of Ethiopia, similar to a ward that consists of at least 3000–5000 people. As a result, a total of seven kebeles and seven health centers were included in this study. The kebeles included in this study were Kuche, Zalema, Tiatia, Ambaye, Shambela, Markuma, and Kentefen. The selected health centers were also Tiatia, Kuche, Alefa, Dereqwua, Shendi, Koki and Wogedade health centers.

### 2.2. Study Design, Participants, and Sampling Procedure

A qualitative descriptive explorative study design was employed to identify and describe factors that influenced or motivated women to give birth in a health facility in their previous, current, and future pregnancies.

Purposive sampling technique was used to select the study participants in this study. The study participants were pregnant women and women who gave birth within one year. Those women who had previously given birth at least once were purposively selected because they had the experience of giving birth in a health facility. Hence, this enabled the researchers to explore and comprehensively understand the factors that influenced or motivated women to utilise skilled attendance at birth. Having oriented the aim of the study and inclusion criteria to the health extension workers (HEWs), who were working in the selected kebeles, the health extension workers identified and recruited the study participants. Furthermore, the researchers corroborated whether they fulfilled the inclusion criteria or not.

### 2.3. Data Collection

Data collection took place between January and February 2016. The data collection team was composed of the researcher and two female research assistants. They were graduates in the fields of health science and sociology, with previous experience of qualitative data collection. The researchers were working in the government health office at the time of the data collection. The researcher organised refresher training on interview skills, data transcription, and management prior to the actual data collection.

The researchers introduced themselves to the participants, explained the purpose of the research, and obtained a written informed consent from each participant before commencement of the actual data collection. A semistructured focus group guide was used to collect data in the current study. The researcher developed a written focus group discussion guide in advance and the guide was very specific to the research questions with carefully worded open-ended questions. The topics of the focus group guide were derived from the literature review, theoretical orientation of the study, and the main research questions of the current study. The focus group guide was composed of open-ended questions that enabled the researcher to know the participant's orientation on the research topic. The open-ended questions in the guide were sequenced flexibly in a pattern of the main question, follow-up questions, and probing questions. The researcher posed the main question to the participants to clarify the idea of the topic or guide the direction the researcher wanted the question to take. The main question posed to the focus group discussion participants in the current study was “describe your perception and experiences with regard to the utilisation of skilled delivery service.” Follow-up questions were asked to take the discussion to a deeper level by asking for more details and these were accompanied by further probing questions to move the discussions to still a deeper territory with or without being specific to the topic of discussion. The focus group guide was prepared in English, translated to Amharic, which is the national working language in Ethiopia, and spoken well in the Amhara region. The focus group discussions were audiotaped and supplemented with notes taken during the discussion. The note taker expanded the notes after each focus group discussion session and shared them among the research team members. This enabled the researcher to devote his full attention to listening to the discussion and probing in-depth information. The audiotape recording provided an accurate, verbatim record of the discussion and captured the language used by the participants in more detail. The researcher sought the informed consent of the participants prior to using the audiotape recording by providing a clear, logical explanation about its use, reassurance about its confidentiality and explained what would happen to the tapes and transcripts. The researchers utilised two audiotape recorders; one was used as a backup in case the other audio tape recorder failed. The researcher tested the scope of the focus group discussion guide, carried out initial tests of the fieldwork, and piloted the focus group discussion guide, as it was a critical part of the research. The pilot testing was conducted a few days ahead of the actual data collection commencement in one district health office, one health centre, and two health posts that were not among the selected study sites for the actual research. The researcher conducted two focus group discussions with pregnant women, and two with women who recently gave birth, and an individual interview with district health office technical officer to pilot test the scope of the guide, fieldwork strategies, and the focus group discussion guide. This enabled the researcher to refine the fieldwork strategies and fine-tune the topic guide by arranging the questions in a logical order, adding or removing minor follow-up questions and estimating the duration of focus group discussions to check for appropriateness of data collection procedures and to familiarize the researcher with the data recording materials such as the audiotape recorder. The focus group discussions were conducted in the health posts that were easily accessible to the study participants and this helped the researchers to avoid any physical and noise nuisance from nonparticipants. The focus group discussions lasted at least sixty to ninety minutes. Focus group discussions with pregnant women and with women who gave birth within one year were conducted separately to make the most out of their shared experiences. A total of 14 focus group discussions were conducted, with pregnant women (7 groups) and women who gave birth within one year (7 groups). Each focus group consisted of 7 to 12 members. The researcher recognised that no new idea or insight emerged after conducting five focus group discussions with pregnant women and five with women who gave birth within one year, which revealed data saturation. An additional two focus group discussions with pregnant women and two with women who gave birth within one year were conducted in order to ensure that data saturation was reached and further data collection stopped at this point.

### 2.4. Data Analysis

The analysis of the data was initiated on the field before the completion of data collection. The researcher listened to the audio files and read the expanded field notes and transcripts after the end of each focus group discussion session and the transcripts were ready to use. This helped the researchers to make the necessary revisions and refinements in the subsequent focus group discussion sessions. The audiotape records of the focus group discussions were transcribed and the research assistants to prepare the interview transcripts for analysis expanded the field notes. The researchers translated the Amharic transcripts directly into English. The researchers' colleague who fluently speaks both English and Amharic checked the consistency between the Amharic transcripts and their English version. The engagement of the researchers in the translation and partly in the transcription of the interviews familiarized and acquainted with the concepts as the researchers read the Amharic transcripts and their English version iteratively in the process.

An inductive thematic analysis approach was employed to analyze the qualitative data. The translated data were exported onto Atlas ti version 7 qualitative data analysis software to efficiently store, organise, manage, and reconfigure the data to enable human analytic reflection. The current study adhered to the following qualitative data analysis steps embracing reading, coding, displaying, reducing, and interpreting.

### 2.5. Ethical Considerations

Ethical clearance was obtained from the University of South Africa (UNISA) Department of Health Studies Higher Degrees Committee and Amhara Regional Health Bureau Research and Laboratory Department to conduct the current study. Letters of support were obtained from all levels of the health system and granted access to the study sites. Written informed consent was taken from participants who could read and write, whereas fingerprints were used to obtain signed informed consent from participants who were unable to read and write. Confidentiality was ensured by removing all names and addresses of participants from the data collecting tools. The information that the participants provided was kept confidential and used only for the purpose of the research. Only codes were used to identify participants, along with audiotape recorders. Anonymity was ensured through the use of codes, thus making it difficult to attribute responses to particular participants. Data collected were kept in the strictest confidence; they were not made public to other people. Audiocassette tapes were also erased after the completion of the study. Only aggregated demographic information was reported to maintain anonymity.

## 3. Results

### 3.1. Basic Sociodemographic Characteristics of the Participants

A total of 133 women participated in the focus group discussions. Of these, 62 women participated in the pregnant women group while the remainder 71 participants included in the women who gave birth within one year category. Of all participants, most of them were in the age group of 20–29 years while about 100% were married and Orthodox Christians. Virtually all participants were unemployed and two-thirds of them never went to school. Furthermore, about half of them got pregnant at least three times. Most of the participants, pregnant women group (62.9%) and mothers delivered within one year group (57.7%), had between 1 and 3 children (Table 1).

In this study, two major themes and a number of subthemes emerged from the focus group discussions with pregnant women and women who gave birth within one year. The major themes were factors that influenced the utilisation of skilled birth attendance service in previous deliveries and factors that would motivate women to utilise skilled delivery care in current and future pregnancy.  Table 2 displays the themes and categories that emerged from analysis of the data.

### 3.2. Influencers to Use Skilled Delivery Care in Previous Childbirth

The participants in this study identified many reasons for the utilisation of skilled delivery care in previous deliveries which included the availability of basic maternal health interventions, fear and experience of obstetric danger signs and complications, and experience of use of services. These factors were subgrouped into categories and subcategories.

#### 3.2.1. Availability of Basic Maternal Health Interventions

The availability of basic and essential maternal health interventions including ambulance transportation service, prevention of mother to child HIV transmission service (PMTCT), referral service, and basic emergency obstetric care identified as factors that influenced women to utilise skilled delivery care in their previous deliveries. Therefore, ensuring the availability of basic maternal health interventions in health facilities could influence women to use skilled birth attendance service.

*(1) Transportation Service*. The participants reported that the availability of ambulance service to transport labouring women from their homes to health facilities was one of the reasons to deliver in health facilities in their preceding deliveries. They also said that the presence of an ambulance transport service was an advocacy tool for the community and enticed women to give birth in the health facility.

The first thing is the presence of ambulance; the second thing is the education we are receiving from health extension workers who influenced us to use skilled birth attendance. (Pregnant woman, FGD1)

The availability of the ambulance service is an educational tool for us and an educational material for us…On top of the health extension workers' health education, the presence of the ambulance was very attractive for the community to give birth in health facilities. (Recently delivered mother, FGD7)

*(2) Prevention of Mother to Child HIV Transmission*. The participant women claimed that the provision of prevention of mother to child HIV transmission service (PMTCT) in health facilities influenced women to utilise skilled birth attendance services in their previous deliveries. Due to the availability of prevention of mother to child HIV transmission service, women sought skilled delivery care from health facilities to prevent the transmission of HIV to the child in case they contracted HIV infection.

One focus group discussion participant corroborated this:

In case we have HIV infection, we give birth in health facility in order to prevent its transmission to the child. Because of this, I had a follow-up visit in the health facility since conception. I decided to go to the health facility regardless of my HIV status. Then, I came to the health facility when I gave birth. (Recently delivered mother, FGD2)

*(3) Referral Service*. The availability of referral service in health facilities also identified as a factor for utilising skilled birth attendance service in their previous deliveries. The participant women reported that they gave birth in health facilities owing to the fact that they could have received referral service if they encountered health problems and/or complications.

If I got sick and got worse, they would refer me to other health facility. They said that if you get sick and get worse, we will refer you to higher health facility. (Recently delivered mother, FGD2)

They will assist us to deliver our child if they can; otherwise they will refer us to the higher health facilities. (Pregnant woman, FGD2)

*(4) Basic Emergency Obstetric Care*. Women reported that the availability of basic emergency obstetric care in health facilities influenced them to utilise skilled birth attendance services in their previous deliveries. The participants claimed that the reasons they chose to deliver in health facilities were that they could have received injections, glucose, and antihypertensive drugs if they experienced haemorrhage, hypertension, and other obstetric complications.

If I had excessive bleeding, there was an injection. That is why I came to the health center. (Recently delivered mother, FGD3)

Intravenous glucose could have been administered whether I had hypertension or anaemia. Anti-hypertensive drugs could have been administered intravenously if I had hypertension; that is why I gave birth in health facilities. (Pregnant woman, FGD3)

#### 3.2.2. Experiencing Obstetric Health Problems

The current study found out that experiencing obstetric danger signs and complications in previous pregnancies and deliveries emanated as factors that influenced women to utilise skilled birth attendance service. Interventions that enhance awareness of women on obstetric danger signs and complications improve utilisation of skilled birth attendance service.

*(1) Experiencing Obstetric Health Problems in Previous Childbirth*. The current study established that experiencing obstetric problems and complications in previous childbirths influenced women to utilise skilled delivery care in subsequent pregnancies.

I went to health facility in my recent delivery because I had excessive bleeding during my first childbirth, but my families advised me against health facility childbirth. Thus, I insisted on to take me to health center because I must go to health center. (Pregnant woman, FGD7)

Furthermore, women claimed that they sought skilled birth attendance service in their recent delivery because they had experienced obstetric danger signs and complications during pregnancy and childbirth. In this regard, many participant women reported that they went for skilled birth attendance service when they experienced prolonged labour at home.

I went to the health center because I had a prolonged labour. I could not give birth at home despite my families repeatedly stew coffee and baked “injera” and then they took me to the health center. I gave birth there and came back to home. (Recently delivered mother, FGD5)

*(2) Perceived Susceptibility to Obstetric Health Problems*. The findings of the current study revealed that women utilised skilled birth attendance service because of fear of the occurrence of unforeseen health problems and obstetric complications resulting if they delivered at home. In addition, fear of perceived obstetric complications sequel due to long years of injectable contraceptive use influenced women's decision to give birth in health facilities.

We had used Injectable contraceptives for many years and this might cause constriction of the birth canal and lead to obstetric complications. So, we feared the complications and visited health facility for skilled delivery care. (Recently delivered mother, FGD1)

If we give birth at home, we may get complicated due to hypertension; we may bleed. Previously, many women died of these complications. Therefore, we delivered in health facility. (Recently delivered mother, FGD7)

#### 3.2.3. Utilisation of Basic Maternal Health Services

This category dealt with the different types of services experienced by women that influenced them to utilise skilled delivery care. This category was subcategorised into counselling service, use of antenatal care, the experience of safe childbirth, and the use of emergency obstetric care.

*(1) Counselling Service*. The participant women claimed that the information and advice they were receiving from health care providers regarding the importance of skilled birth attendance service, the various types of services rendered during health facility delivery, and about the risks associated with home delivery influenced them to utilise skilled birth attendance service. Therefore, provision of good counselling service on the benefits of skilled delivery care, services provided in health facility childbirth, and dangers associated with home delivery influence women to utilise skilled delivery service.

During antenatal care visits, health workers advised me to deliver my baby in the health center and they told me not to give birth at home. I said okay. I accepted their advice when they told me the risks associated with home delivery. (Recently delivered mother, FGD6)

The issues that influenced us to utilise skilled birth attendance service are; firstly, the health extension workers gave us health education with close follow-up. (Pregnant woman, FGD6)

*(2) Use of Antenatal Care*. Women reported that having regular antenatal care follow-up during pregnancy influenced them to utilise skilled birth attendance service during childbirth. They said that they had received information and advice on maternal and newborn health and different preventive and treatment services during antenatal care which in turn made them seek skilled delivery care.

Since we got pregnant, the health workers told us to come and have an examination; we came and got examined. When we had a monthly antenatal care check-up, they told us to come to the health facility when our expected date of delivery approached. (Recently delivered mother, FGD3)

For instance, we had a regular monthly antenatal care follow-up visits in the health center and health post. The health workers were providing counselling services by gathering pregnant women about the risks of giving birth at home and advise us against home delivery; they provided us health education. After that, the health workers closely followed us even by visiting to our home. It is because of their education the community is utilising skilled delivery service. (Recently delivered mother, FGD6)

*(3) Safe Childbirth Experience*. Ensuring the wellbeing of the parturient women and their neonates was found out to be a factor that influenced women to deliver in a health facility. The participants accounted that they had received close follow-up, care, and encouragement from health workers during health facility childbirth. Hence, they gave a safe birth in health facilities and their neonates also received close follow-up and care. The parturient women also noted that they and their neonates got examined and treated for any health problems and disease conditions to ensure the welfare of the mothers and neonates.

The health workers immediately followed us and they encouraged us in every aspect. Nothing bad happened to us and we gave birth safely; in addition, our newborns were fine and received close follow-up; we went back to our home safely so that giving birth in a health center is a very good thing. (Pregnant woman, FGD7)

*(4) Use of Emergency Obstetric Care*. The current study revealed that the various medical interventions received by women to detect and manage unforeseen obstetric problems and complications influenced them to utilise skilled birth attendance services. The participants believed that they did not encounter any obstetric problems and complications during childbirth because they delivered in health facilities. Hence, they received various medical interventions during labour and childbirth to detect and manage obstetric health problems and complications.

We delivered in health facilities to protect ourselves from experiencing any obstetric complications. (Recently delivered mother, FGD3)

Now, nobody dies of pregnancy and childbirth because we are giving birth in health center. Therefore, we do not experience any complications like hypertension, bleeding. We give a safe birth in health center and we go back to our home. (Pregnant woman, FGD5)

### 3.3. Motivators to Use Skilled Delivery Care in Future Childbirth

This theme dealt with factors that would motivate women to seek skilled delivery care in future childbirth. This theme comprised of six categories, namely, good interpersonal care, availability of basic maternal health services, previous childbirth experience, ensuring welfare of parturient women and newborns, and perceived susceptibility to obstetric health problems.

#### 3.3.1. Good Interpersonal Care

The study participants narrated that good interpersonal care from maternity care providers would motivate women to utilise skilled delivery care in future childbirth which includes good counselling service and respectful and caring maternity care providers.

*(1) Good Counselling Service*. The participant women reported that the counselling and advice on maternal and newborn health they were receiving from maternity care providers would motivate them to utilise skilled birth attendance services in future pregnancies.

If I get pregnant in the future, I will give birth in the health center. What encourages me to deliver in the health center; their advice; their advice during my monthly antenatal care follow-up; their advice is enough for me. (Recently delivered mother, FGD1)

They will advise us about newborn care. Previously, butter was given for the newborn to swallow and put on the cord. Thus, they will advise us not to practice such things. In order to know these things, we should give birth in health facility. (Pregnant woman, FGD7)

*(2) Respectful and Caring Maternity Care Providers*. The presence of respectful and caring maternity care providers in health facilities would motivate the women to utilise skilled birth attendance services in their future pregnancies. The findings indicated that friendly service and close follow-up from maternity care providers would encourage them to utilise skilled birth attendance services.

I would like to give birth in a health facility because we receive health education. Firstly, even our father and mother do not care us like that and we give birth there. There is a coffee ceremony and porridge preparation; besides, they also provide us clothes. We feel like as if we did not have any labour pain. (Recently delivered mother, FGD7)

#### 3.3.2. Availability of Basic Maternal Health Interventions

The current study established that the availability of ambulance transportation service, referral service, and women friendlily service would motivate women to utilise skilled birth attendance services in their future pregnancies.

*(1) Ambulance Transportation Service*. The focus group discussion participants recounted that the availability of ambulance services for labouring women would motivate women to utilise skilled birth attendance services in future pregnancies. Hence, availing transportation services for labouring women would spur them to give birth in health facilities.

I am planning to give birth in health facility. I have already received the phone number of the ambulance. (Pregnant woman, FGD4)

We can call for the ambulance service and the ambulance will come to us. So that, I will deliver in health facility. (Recently delivered mother, FGD6)

*(2) Availability of Referral Service*. The participant women indicated that the availability of referral services would encourage women to utilise skilled birth attendance services in their future pregnancies. They claimed that they could use referral services in circumstances where they faced health problems and obstetric complications.

I will give birth in this health center…In case the placenta might come out ahead of the foetus, I would like to take referral from this health center to other higher health facility. (Pregnant woman, FGD3)

*(3) Women Friendly Services*. This study indicated that the availability of cultural ceremonial practices such as coffee stewing, porridge cooking, and provision of clothes for newborn in health facilities would motivate women to utilise skilled birth attendance services in the current and future pregnancies. Furthermore, the provision of women friendly services would also encourage women to utilise skilled birth attendance services in the current and future pregnancies.

I want to give birth in health center because…there is a coffee ceremony; and they are also providing cloths for newborns. I heard that they encourage women to give birth in health facilities through coffee ceremony; they also provide clothes for newborns. (Recently delivered mother, FGD5)

#### 3.3.3. Previous Childbirth Experience

The study showed that experiencing obstetric health problems in previous home delivery and health facility childbirth in previous delivery would motivate women to use skilled birth attendance service in the current and future pregnancies.

*(1) Experiencing Obstetric Health Problems in Previous Home Delivery*. The study participants voiced that they experienced health problems and complications when they previously gave birth at home. In consequence, the parturient women and their neonates suffered a lot from these health problems and obstetric complications and they decided to seek skilled delivery care in future pregnancies. Therefore, the current study revealed that experiencing health problems and childbirth related difficulties in previous home deliveries would make women recognise the use of skilled birth attendance services in the current and future pregnancies.

Now, I am thinking that I will go to the health center and give birth there as soon as my labour starts. Previously, I gave birth at home…I had suffered a lot and my labour persisted for more than 12 hours. In addition, inexperienced individuals attended my delivery as a result they over stretched the umbilical cord and her umbilicus is still bulged. Hereafter, I will inform the health extension worker as soon as my labour starts and the health extension worker will call to the ambulance and transport me to the health facility. (Pregnant woman, FGD6)

*(2) Health Facility Childbirth in Previous Delivery*. The participant women purported that they intended to give birth in health facility in the current and future pregnancy because they gave birth safely in their previous health facility deliveries and bearing in mind that it would be the same as the previous health facility childbirths. Hence, the utilisation of skilled birth attendance services in previous deliveries would encourage women to do so in the current and future pregnancies, too.

I would like to give birth in the health center. Because I gave birth safely in the health center in my previous delivery and now we think it would be the same us the previous delivery so that I would like to utilise skilled birth attendance service. (Recently delivered mother, FGD2)

Since I gave birth in health facility previously, I am thinking now to come and give birth here in the health center. (Pregnant woman, FGD3)

#### 3.3.4. Ensuring Welfare of the Parturient Women and Newborns

The study participants pointed out that they intended to use health facility childbirth to ensure the wellbeing of the women and their neonates. Under this category, disease prevention and treatment services, safe childbirth, and access to emergency obstetric care were identified as motivating factors to utilise skilled birth attendance service in the current and future pregnancies.

*(1) Prevention and Treatment of Disease Conditions*. The participant women claimed that they would utilise skilled birth attendance services in the current and future pregnancies in order to get preventive and treatment services for disease conditions.

I will give birth in the health facility. Because they will provide us different preventive services to protect us from different diseases. (Recently delivered mother, FGD3)

For instance, there could be a woman who is infected with HIV. When she gives birth, HIV can be transmitted from the mother to child during childbirth. The drug is being provided in the health center. If the woman does not receive the drug from the health center, the newborn also could get infected. (Recently delivered mother, FGD2)

*(2) Safe Childbirth*. The women claimed that they were intending to utilise skilled birth attendance service in the current and future pregnancies in order to ensure the health of the mother and the newborn. Besides, the parturient women and newborns would receive proper care and follow-up in health facility childbirth. For instance, the neonates could receive proper thermal and cord care and the women would be protected from experiencing obstetric complications such as excessive bleeding.

The newborn would be put on heater to protect the baby from cold and the cord would be cut using a clean material. (Recently delivered mother, FGD7)

I will give birth in the health facility. Because they would help us. They care for both the newborn and me. I would give birth safely and go back to my home. (Pregnant woman, FGD3)

I would like to give birth in health facility because I do not experience excessive bleeding and the newborn would be healthy. I am planning to give birth in health facility in the future. (Recently delivered mother, FGD4)

*(3) Availability of Emergency Obstetric Care*. The study participants claimed that they would like to give birth in health facilities in the current and future pregnancies because they could receive emergency obstetric care in cases of excessive bleeding, anaemia, prolonged labour, and other obstetric complications befall to women. Hence, the availability of early detection and management of obstetric health problems and complications service in the health facilities could influence women to utilise skilled birth attendance services in the current and future pregnancies.

What motivate us to utilise skilled birth attendance service…in case the woman encounters bleeding, she would be given injections. (Pregnant woman, FGD4)

I will give birth in the health center in order to avert many obstetric problems including bleeding, anaemia, and post-partum haemorrhage. The newborn and I will have a safe and clean delivery. (Pregnant woman, FGD6)

#### 3.3.5. Perceived Susceptibility to Obstetric Health Problems

Fear of occurrence of obstetric danger signs and complications could motivate women to utilise skilled birth attendance service in the current and future pregnancies. The participant women indicated that they would like to give birth in health facilities in the current and future pregnancies since they had been using modern family planning methods for a long time. Therefore, they perceived that their birth canal might get constricted and, as a result, they feared they could develop obstetric danger signs and complications.

I would like to give birth here. Because I had been using Injectable contraceptives for long time, I expect that my labour might get prolonged. (Pregnant woman, FGD1)

I am thinking to give birth in health facility. It has been long time since I gave birth and I had been using family planning so that I am planning to go to health facility for childbirth and see there. (Pregnant woman, FGD2)

## 4. Discussion

Our study set out to make an in-depth exploration to identify factors that influenced or would motivate women to give birth in a health facility in the previous and future pregnancies in North West Ethiopia.

Our study reveals that access to basic services such as ambulance transport service, prevention of mother to child HIV transmission service, referral service, women friendly service, and lifesaving interventions influenced women to utilise skilled birth attendance service in the previous and subsequent deliveries. In this regard, the availability of transportation service [23–25], prevention of mother to child HIV transmission service [26], referral service [24], women friendly service [27], and lifesaving interventions [23, 28] were critical aspects of maternal health, highly valued by women, and associated with better maternal health utilisation. Therefore, improving access to such basic services for women enhances the utilisation of skilled delivery care.

The current study found that the experiences of encountering obstetric health problems and complications in previous childbirths influenced women to utilise skilled birth attendance service in their follow-up on pregnancies. This finding is consistent with studies conducted in low and middle income countries [29, 30] which concluded that previous pregnancies and delivery complications found to be significant predictors of the utilisation of skilled birth attendance service. In this study, women also utilised skilled birth attendance service because of fear of the occurrence of unanticipated health problems and obstetric complications resulting from home delivery and the sequel of many years of using injectable contraceptives. A study conducted in North West Ethiopia [30] reported that family planning users were more likely to utilise skilled birth attendance services as compared to those who did not. Besides, women who had previously given birth at home would prefer to have institutional delivery for the next child, as health care providers secure at the facilities as there was the assurance of appropriate medical care in case of any emergency [23].

Moreover, the findings of the current study revealed that experiencing health problems and childbirth related difficulties in previous home deliveries would motivate women to utilise skilled birth attendance service in the current and future pregnancies. A study conducted in Northern Ethiopia showed that previous negative home delivery experiences when women faced life threatening complications, made them recognise medical services in their next pregnancies and childbirth [28].This finding is in line with a qualitative synthesis of studies in low and middle income countries which indicated that previous delivery experiences or birth outcomes would inform their future delivery location. A woman is more likely to give birth in a health facility if she had negative previous delivery experiences or birth outcomes [30].

The study also indicated that the information and advice provided by health care providers, having regular antenatal care follow-up during pregnancy, use of emergency obstetric care, and ensuring the wellbeing of the parturient women and their neonates were found out to be the factors that influenced women to give birth in health facility. This finding was corroborated by several studies done in Africa [23, 28, 31, 32].

Good interpersonal care from health care providers would motivate the women to utilise skilled birth attendance service in their future pregnancies. It was indicated that a friendly service and close follow-up from health workers would encourage them to utilise skilled birth attendance service. In this regard, the presence of health care providers in health facilities who are respectful, caring, friendly, helpful, and sympathetic was an important factor in encouraging the demand, utilisation of maternal health care, and critical component of good skilled delivery care [23, 24, 33].

The utilisation of skilled birth attendance service in previous deliveries would also motivate women to use skilled birth attendance service in the current and future pregnancies. Women who previously delivered with a skilled attendant would become more familiar with the medical setting, which would make them more likely to use it again [17, 32, 34].

In this study, women claimed that they would utilise skilled birth attendance service in the current and future pregnancies in order to get preventive treatment services for disease conditions. Mahiti et al. [35] and Sword et al. [36] corroborated this finding in their studies that the women recognised the importance of skilled birth attendance services as they provided various investigations and treatment of diseases such as malaria, HIV, and preexisting health conditions.

## 5. Conclusion and Recommendation

Although the institutional delivery rate in Ethiopia has shown improvement over a period of time, evidence showed that the proportion of births attended by a skilled birth attendant was very low. Several studies conducted in Ethiopia identified multitudinous influencing or motivating factors for health facility childbirth though a large body of evidence derived from quantitative researches. The current study identified factors that influenced or motivated women to utilise skilled delivery care in the previous, current, and follow-up on pregnancies in North West Ethiopia. In this regard, access to basic and essential services such as ambulance transport service, prevention of mother to child HIV transmission service, referral service, and emergency obstetric care, fear and experience of obstetric danger signs and complications, reception of information and advice on health issues, use of antenatal care, use of emergency obstetric care, and safe childbirth experiences were identified as factors that influenced the utilisation of skilled delivery care in previous pregnancies. Furthermore, the study identified factors that would motivate women to seek skilled delivery care in future childbirth. Likewise, the availability of ambulance transport service, referral service, emergency obstetric care, and women friendly service would motivate women to utilise skilled delivery care in future pregnancies. The presence of caring and friendly maternity care providers, good counselling service, experience of health problem and complication in previous home delivery, use of skilled delivery care in previous childbirth, ensuring wellbeing of parturient women and newborns, and fear of obstetric danger signs and health problems were also reported to be the factors that would motivate the utilisation of skilled birth attendance service in subsequent pregnancies.

### 5.1. Recommendations

1. Accessing basic services including ambulance transport service, robust referral service, emergency obstetric care, prevention of mother to child HIV transmission service, and women friendly service including the practice of cultural ceremonies improve the utilisation of skilled birth attendance service. Therefore, it is recommended that health facilities need to make sure that these services are offered for women around the clock.
2. Improving the coverage of focused antenatal care service and its utilisation by women helps to improve the utilisation of skilled delivery care, therefore, encouraging women to use focused antenatal care and practice birth plan and complication readiness to improve health facility childbirth and manage obstetric health problems and complications.
3. Health facilities should improve the interpersonal care between health workers and women embracing good counselling service and the presence of respectful and caring maternity care providers.
4. Health facilities should develop and/or implement information education communication (IEC)/social behavioural change communication (SBCC) interventions and tools, provide health education, and undertake advocacy and social mobilisation to improve awareness of the community on the importance of skilled birth attendance service, risks of home delivery, and obstetric danger signs and complications.
5. Health facilities should provide basic emergency obstetric and newborn care to deal with obstetric health problems and complications.
6. Health facilities need to be organised in a way that provides women centered care. It became evident that an enabling environment should be set up in health facilities to allow parturient women and their families or relatives to practice cultural ceremonies.

## Figures and Tables

**Table 1:** Demographic characteristics of participants.

Characteristics		Women who gave birth within one year (*n* = 71)		Pregnant women (*n* = 62)	
		Frequency	Percentage	Frequency	Percentage
Age	17–19	3	4.2	1	1.6
	20–24	20	28.2	16	25.8
	25–29	27	38.0	22	35.5
	30–34	16	22.5	17	27.4
	≥35	5	7.0	6	9.7

Religion	Orthodox	71	100	62	100

Marital status	Divorced	3	4.2	0	0.0
	Married	68	95.8	62	100

Occupation	Employed	1	1.4	1	1.6
	Unemployed	70	98.6	61	98.4

Education	No education	46	64.8	40	64.5
	Primary	10	14.1	7	11.3
	Secondary	7	9.8	7	11.3
	High school and above	8	11.3	8	12.9

Gravidity	1–3	35	49.3	31	50.0
	4–6	31	43.7	28	45.2
	≥7	5	7.0	3	4.8

Parity	0	0	0.0	8	12.9
	1–3	41	57.7	39	62.9
	4–6	27	38.0	14	22.6
	≥7	3	4.2	1	1.6

**Table 2:** Themes and categories that emerged.

Themes	Categories
Influencers to use skilled delivery care in previous childbirth	(i) Availability of basic maternal interventions (a) Basic emergency obstetric care (b) Referral service (c) Prevention of mother to child HIV transmission service (PMTCT) service (d) Ambulance transport service (ii) experiencing obstetric health problems (a) Experiencing obstetric health problems in previous childbirth (b) Perceived susceptibility to obstetric health problems (iii) Utilisation of basic maternal health services (a) Counselling service (b) Use of antenatal care (c) Safe childbirth experience (d) Use of emergency obstetric care

Motivators to use skilled delivery care in future childbirth	(iv) Good interpersonal care (a) Counselling service (b) Respectful and caring maternity care providers (v) Availability of basic maternal health services (a) Ambulance transportation service (b) Referral service (c) Women friendly services (vi) Previous childbirth experience (a) Obstetric health problems in previous home delivery (b) Health facility childbirth in previous delivery (vii) Ensuring the welfare of the parturient women and newborns (a) Prevention and treatment of disease conditions (b) Safe childbirth (c) Access to emergency obstetric care (viii) Perceived susceptibility to obstetric health problems

## Data Availability

The data used to support the findings of this study are available from the corresponding author upon request.
